# Utilization of institutional delivery service and associated factors among mothers in North West Ethiopian

**DOI:** 10.1186/s13104-018-3295-8

**Published:** 2018-03-27

**Authors:** Solomon Weldemariam, Amare Kiros, Mengistu Welday

**Affiliations:** 1 Department of Midwifery, College of Health Sciences, Mekelle University, Mekelle, Ethiopia; 2 Department of Midwifery, College of Health Sciences, Pawe, Ethiopia

**Keywords:** Institutional delivery, Associated factors, Benishangul-Gumez, Ethiopia

## Abstract

**Objective:**

The aim of this study was to assess institutional delivery and its associated factors in Benishangul-Gumez region, North-West of Ethiopia. The data were obtained at community level in a single survey within 1 month and there is no continuation of this study or previously published part elsewhere.

**Results:**

Among the 428 eligible respondents recruited for this study, 427 of them responded completely to the interview, giving a response rate of 99.8%. Of the total (427) respondents, 51.1% women delivered the recent child at health facility in the 12 months preceding the survey. Among the common reasons for home delivery were, labour was urgent (25.8%), home birth was usual habit for them (23.9%) and distance to health center was too far. Age (AOR = 3.4, 95% CI 1.46, 7.97), husband occupation (AOR = 5.16, 95% CI 1.74, 15.31), frequency of antenatal care visit (AOR = 3.34, 95% CI 1.88, 5.94) and maternal knowledge on danger signs of pregnancy and delivery (AOR = 7.18, 95% CI 3.77, 13.66) were significantly associated factors with institutional delivery. Although, the prevalence of institutional delivery has improved when compared to previous reports, strategic modification is important to increase health facility delivery.

**Electronic supplementary material:**

The online version of this article (10.1186/s13104-018-3295-8) contains supplementary material, which is available to authorized users.

## Introduction

Globally, the total number of maternal deaths decreased by 45% from 523,000 in 1990 to 289,000 in 2013 with an annual rate of decline by 3.3% [1]. In sub-Saharan Africa (SSA), a woman’s risk of dying from preventable complications of pregnancy and childbirth is 1 in 22 [2]. The 99% (286,000) of the global maternal deaths occurred in developing countries, SSA region alone accounting for 62% (179,000) of these deaths [1]. Ethiopia is among countries with highest maternal mortality ratio (MMR) in the world with an estimated MMR of 676/100,000 live births and it is one of among the ten countries accounted for 58% of the global maternal deaths in 2013 [1, 3].

Though skill-attendance is the most important intervention to prevent life threatening complications [4, 5], most SSA women still give birth at home in the absence of skilled birth attendant (SBA) [4]. Worldwide, the major direct causes of maternal mortality are haemorrhage, sepsis, unsafe abortion; pregnancy induced hypertension and obstructed labour [6]. Haemorrhage alone accounts for one-third of all maternal deaths in Africa [7]. Wide disparities are found among regions in the level of health facility delivery ranging from nearly universal in Western to about 50% in Southern Asia and SSA [8]. Socio demographic variables, birth order, distance to health facility, exposure to media, frequency of antenatal care (ANC), knowledge on pregnancy and child birth danger signs, history of prolonged labour, and decision making status are among the major factors cited for low uptake of institutional delivery [4, 9–13].

Ethiopia in its’ Health Sector Development Plan IV’s targeted to increase SBA to 62% by 2015; but the coverage has reached 55% [14]. Literatures from Ethiopia also reported that institutional delivery ranges from 15 to 47% [11, 15]. So as to compensate this gap, Ethiopia in its health sector transformation plan has set a goal to increase SBA to 90% by 2019/20 [16]. Factors associated with institutional delivery appear to be context related and vary across ranges of studies in Ethiopia [9, 10, 17–19]. Therefore, it is a crucial point to identify contextual factors determining institutional delivery to help policy maker as guide for possible context based strategic modification of programs and interventions. Moreover, the study area is an emerging region and studies done in this area are limited.

## Main text

### Methods

#### Study setting

This study was conducted in Pawe district one of among the districts of Benishangul-Gumez Regional state in Ethiopia. Pawe district has 20 kebeles. Based on the information obtained from the district health office, the population of Pawe district was about 57,724 in 2014 of which, 13,882 were reproductive age group women (unpublished report). Community based cross sectional study was conducted from March 1 to March 30, 2015.

#### Sample size and sampling techniques

A single population proportion formula [n = (Z α/2)^2^ p(1 − p)/d2] was used to estimate the required sample size. The following assumptions were made while calculating the sample size. A 95% CI, 5% (d = 0.05) margin of error, population proportion of mothers who gave birth at health institution assumed to be 15.8% [15], design effect of 2 with an expectation of 10% of non-response rate. Overall, 428 respondents were recruited. The kebeles found in the district (17 rural kebeles and 3 urban kebeles) were stratified into rural and urban strata then lottery method was used to select five kebeles from the rural strata and one kebele from the urban strata to ensure representativeness. Proportional to population size allocation technique was used to allocate the sample size to each kebele. Census was done in each kebeles to identify households with eligible women and corresponding identification number was given to develop sampling frame. Then systematic sampling technique was used to recruit eligible respondents at every kth interval (k = 2, 3).

#### Inclusion and exclusion criteria

The study population included all women who delivered within the last 12 months preceding the survey and residing in the selected kebeles at least for 6 months.

#### Data collection tool and procedures

A structured questionnaire was developed from different literatures in English and translated into ‘Amharic’ language (local language). Data were collected by six trained diploma midwives who can speak Amharic language through face to face interview. A pretest was conducted among 5% of the sample size in nearby district which has similar basic socio-demographic characteristics as the study district. The overall supervision was carried out by the principal investigator and supervisors. Ethical clearance was obtained from Institutional Ethical Review Board of Mekelle University, College of Health Sciences. A letter of permission was obtained from Metekel zonal Health Office to Pawe district Health Office then to the respective kebeles. Informed signed consent was obtained from study participants and consent for participants below 18 years old was taken from their father/mother.

#### Analysis

Data were checked and entered into Epi Info version 3.3.2 software, and exported to SPSS version 20 software for analysis. Variables with p < 0.2 at bivariate analysis were included in multivariable logistic regression analysis. Odds ratio along with 95% CI was computed to ascertain association between independent and dependent variables. p value of < 0.05 in the multivariable analysis was considered as cut off point to determine statistical test.

### Results

#### Socio-demographic characteristics

Among the 428 eligible respondents recruited, 427 of them responded completely to the interview, giving a response rate of 99.8%. Majority, 318 (74.5%) of respondents were rural residents. The mean age of the respondents was 29.8 years (SD 7.4) with a range of 16–48 years old. The majority of respondents, 381 (89.2%) were married. About, 127 (29.7%) of respondents were had radio or television at home (Table 1).

**Table 1:** Socio-demographic characteristics of the study participants in Pawe district, Benishangul-Gumez, Ethiopia March 2015

Variable	Frequency N = 427 (%)
Religion	
Orthodox Christian	202 (47.3)
Muslim	131 (30.7)
Catholic	40 (9.4)
Protestant	36 (8.4)
Traditional	18 (4.2)
Ethnicity	
Amhara	266 (62.3%)
Hadya	44 (10.3%)
Oromo	43 (10.1%)
Agew	33 (7.7%)
Kembata	24 (5.6%)
Others (Welayta, Tigrian and Gumez)	17 (4%)
Occupation	
Farmer	183 (42.9%)
House wife	177 (41.5%)
Government employee	28 (6.6%)
Others (student, daily laborer, merchant, and private employee)	39 (9.0%)
Husband’s educational status (n = 381)	
Unable to read and write	161 (42.3%)
Primary	111 (29.1%)
Secondary	54 (14.2%)
College or University	55 (14.4%)

#### Reproductive characteristics of respondents

Two hundred seventy (60.9%) respondents were experienced first birth before the age of 20. The mean age at first pregnancy was 19.09 years old (SD 3.017). Twenty (4.7%) respondents had history of still birth. Two hundred thirty-eight (55.7%) respondents were Para two, 119 (27.9%) primi-para and the rest grand multipara (5 +). Three hundred fifty-two (82.4%) of respondents had ANC follow up.

#### Utilization of institutional delivery service

**Fig. 1:**
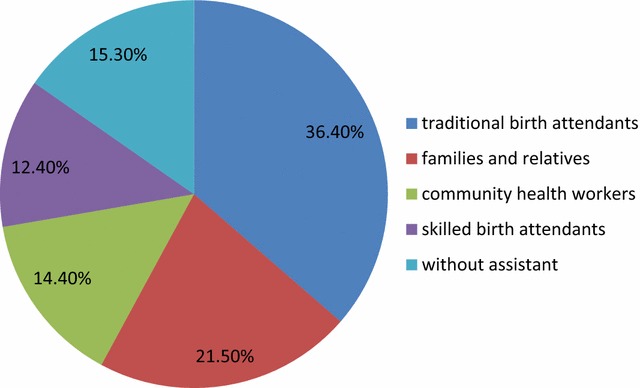
Percentage distribution of birth attendant for women who gave birth at home in Pawe district, Benishangul Gumez, Ethiopia, March 2015

Of the 427 respondents, 218 (51.1%) of them gave birth at health facility. Among women who delivered at health facility, 107 (49.1%) deliveries were in hospital, 77 (35.3%) in health center and 34 (15.6%) in health posts., The common reasons for home delivery were labour was urgent (25.8%), home birth was usual habit for them (24%), health center was too far (19.1%), family influence to deliver at home (14.8%), lack of transportation (9.1%) and no problem at the time of delivery (7.2%). About 15.3% of the home births were delivered without assistant (Fig. 1).

#### Factors associated with institutional delivery

Mothers with age group of less than 25 years old were about 3.4 times (AOR = 3.4, 95% CI 1.46, 7.97, p = 0.005) more likely to deliver in health facility than mothers with age group 35 and above. Mothers whose husbands occupation was governmental employee were 5.2 times (AOR = 5.16, 95% CI 1.74, 15.31, p = 0.003) more likely to deliver in health facility than mothers whose husbands were farmer by occupation. Having 4 + frequency of ANC visit in the recent pregnancy were about 3.3 times (AOR = 3.34, 95% CI 1.88, 5.94, p = 0.001) more likely to give birth in health facility when compared to mothers who had less than 4 frequency of ANC visit. Mothers who were knowledgeable on obstetrical danger signs of pregnancy and delivery were about 7.2 times (AOR = 7.18, 95% CI 3.77, 13.66, p = 0.001) more likely to deliver in health facility when compared to mothers who were not knowledgeable (Table 2).

**Table 2:** Bivariate and multivariable analysis of factors associated with institutional delivery in Pawe district Benishangul-Gumez, Ethiopia March 2015

Variable	Institutional delivery (N = 427)		COR (95% CI)	AOR (95% CI)
	No	Yes		
Age of the mother				
15–24	38	71	3.322 (1.941, 5.684)	3.412 (1.461, 7.972)*
25–34	91	102	1.993 (1.256, 3.162)	1.245 (.636, 2.438)
≥ 35	80	45	1	1
Residence				
Rural	166	152	1	1
Urban	43	66	1.676 (1.077, 2.610)	0.460 (0.176, 1. 201)
Mother’s educational status				
Unable to write and read	129	70	1	1
Primary education	65	85	2.410 (1.560, 3.722)	1.199 (0.646, 2.223)
Secondary and above	15	63	7.740 (4.107, 14.588)	1.233 (0.476, 3.190)
Husband’s occupation (N = 381)				
Farmer	162	124	1	1
Merchant/private employee	9	23	3.339 (1.492, 7.470)	1.519 (.535, 4.317)
Government employee	10	53	6.924 (3.387, 14.155)	5.162 (1.740, 15.310)*
Possession of radio or television				
No	172	128	1	1
Yes	37	90	3.269 (2.093, 5.105)	1.575 (0.786, 3.158)
Last pregnancy planned?				
No	42	19	1	1
Yes	167	199	2.634 (1.475, 4.703)	1.440 (0.604, 3.432)
Time to reach to the nearest HF on walk				
< 30	51	85	1.980 (1.305, 3.004)	2.049 (.887, 4.733)
≥ 30	158	133	1	1
Frequency of ANC visit				
< 4	79	53	1	1
4 +	60	160	3.975 (2.516, 6.280)	3.341 (1.879, 5.939)**
Knowledge on danger signs				
No	120	25	1	1
Yes	89	193	10.409 (6.321, 17.140)	7.176 (3.770, 13.658)**

* Significantly associated at p < 0.05

** Significantly associated at p < 0.001

### Discussion

According to this study, institutional delivery with skilled birth attendant was 51.1%. This indicates that nearly half of mothers were delivered at home without the help of SBA to rescue the life in emergency situation. The current finding is consistent with the findings reported from SSA, Nepal, East Delhi India, and Ethiopia, where the proportion of mothers who gave birth in health facility were 53, 48, 51, 47 and 48.3% respectively [3, 11, 12, 20, 21]. However, the current prevalence of health facility delivery is higher when compared to reports from districts of Ethiopia, where the proportion of mothers who gave birth at health facility were 15.7, 37.9 and 25% respectively [17, 22, 23]. This discrepancy might be due to the difference in intervention that has been taking place by the midwives, Health extension workers and women development army in auditing and mobilizing pregnant mothers for maternal health service utilization to reach all segments of the population.

On the contrary, the current report is lower when compared to reports from districts of Ethiopia, where 97, 61.6 and 62.2% of mothers gave birth in health facility respectively [24–26]. This could be due to the fact that majority of respondents in this study were rural residents, where as participants of the above studies were urban residents. Hence, they could have better decision making autonomy and better access to information than rural residents.

Respondents in the age group of less than 25 years old were about 3.3 times more likely to give birth in health facility when compared to mothers in the age of 35 and above. This finding is consistent with the reports from districts of Ethiopia, where respondents within the age group of 15–24 years were about 4 times more likely to give birth in health facility as compared to age group of 35 and above respectively [18, 22, 27]. This might be due to the fact that younger mothers are more likely to be educated and they may have a better opportunity to access information as compared to older mothers. Respondents whose husbands occupation were governmental employee were about 5.2 times more likely to give birth at health facility as compared to mothers whose husbands were farmers by occupation. This finding is in line with the findings from Bangladesh and Ethiopia [28, 29]. This might be people working in government organizations usually educated and might have better opportunity to access information and use health services when compared to farmers. Respondents who had 4 + frequency of ANC visit were about 3.3 times more likely to give birth in health facility when compared to mothers with less than four visit. This finding is consistent with many reports from parts of Ethiopia [17, 26]. This might be ANC can provide an opportunity for health workers to promote health facility delivery and to counsel birth preparedness and complication readiness. Moreover, mothers who present for ANC follow up have already demonstrated readiness to use available services. Maternal knowledge on danger signs of obstetrics in pregnancy and delivery was about 7.2 times more likely to give birth in health facility as compared to their counterparts. The current finding is consistent with the reports from Ethiopia (Metekel and Sodo) [26, 30]. This might be due to that respondents who had knowledge on danger signs could have a greater fear of complications, lead them to seek skilled attendance during birth.

### Conclusion and recommendation

Although, the prevalence of institutional delivery is high when compared to previous reports from districts of Ethiopia, still institutional delivery is low in the study area. This finding is a clue to modify strategies and programs to close the gaps and improve health service utilization. The interventions that have been taking place by ministry of health to increase health service utilization should be strengthening. Moreover, midwives should strengthen the counseling session regarding pregnancy and delivery danger signs. Midwives should give emphasis in counseling mothers with age group of 25 and above that all pregnancy is at risk of complication and the importance of health facility delivery. Government should give emphasis on strategies that create awareness on farmers to involve in every maternal health care service given for their wives.

## Limitations

This study was conducted in one district of Benishangul-Gumez region therefore the findings might not be generalizable to the entire region. The cross-sectional nature of the study does not allow for causality inferences. Despite of these limitations, being a community based study and its sample size adequacy might be taken as strength.

## Additional files

**Additional file 1.** This SPSS template contains that data that support the findings of this study.

**Additional file 2.** It is an English version questionnaire used to measure this findings and it was developed from different literatures and adjusted contextually by consulting seniors.

## Abbreviations

ANC: antenatal care
DHS: Demographic and Health Survey
EDHS: Ethiopian Demographic and Health Survey
MMR: maternal mortality ratio
SBAs: skill birth attendants
SSA: sub Saharan Africa
TBAs: traditional birth attendants
