# Supplementary material for: Utilization of institutional delivery service and associated factors among mothers in North West Ethiopian
Source: BMC Res Notes. 2018 Mar 27;11:194. doi: 10.1186/s13104-018-3295-8 (PMC5870376; doi:10.1186/s13104-018-3295-8)
Supplement: Supplementary file 2 — Additional file 2. It is an English version questionnaire used to measure this findings and it was developed from different literatures and adjusted contextually by consulting seniors. [file 13104_2018_3295_MOESM2_ESM.docx]

## English version questionnaire

Mekelle University, College of Health Science, Department of Midwifery Survey Questionnaire, to assess institutional delivery and associated factors among mothers who gave birth in the last 12 months in Pawe district Benishangul-Gumez, northwest Ethiopia, 2015

**Part I: Socio demographic characteristics of respondents**

| **S. NO** | **Questions** | **Response** | **Skip pattern** |
| --- | --- | --- | --- |
| 101 | Age | In years--------------- |  |
| 102 | Residence | 1. Rural  2. Urban |  |
| 103 | Marital status | 1. Married  2. Divorced  3. Widowed  4. Single  5. Separated |  |
| 104 | Religion | 1. Orthodox  2. Muslim  3. Protestant  4. Catholic  5. Others(specify)______ ­­­­­­­ |  |
| 105 | Ethnicity | 1. Amhara  2. Kembata  3. Hadya  4. Oromo  5. Agew  8. Others, specify ________ |  |
| 106 | Occupation status | 1. House wife  2. Governmental Employee  3. Private Organization Employee  4. Farmer  5. Merchant  6. Daily Laborer  7. Student  8. Others, specify __________ |  |
| 107 | Educational Status | 1.Unable to read and write  2. Able to read and write  3. Primary education(1-8)  4. Secondary education(9-12)  5. College or University |  |
| 108 | Husband’s educational Status | 1.Unable to read and write  2. Able to read and write  3. Primary education(1-8)  4. Secondary and above (9-12)  5. College or University |  |
| 109 | Husbands occupation | 1. Farmer  2. Governmental employee  3. Private organization Employee  4. Merchant  5. Daily laborer  6. Other, specify___________ |  |
| 110 | Do you have any of the following means of communication? | 1. Radio  2. TV  3. None |  |
| 111 | Time taken to reach the nearby health center on foot in hours? | In hours _____________ |  |

**Part II: Obstetric characteristics of the respondent and place of delivery**

| 201 | | What was your age at first birth | In years--------------- |  |
| --- | --- | --- | --- | --- |
| 202 | | How many times you have pregnant including abortion |  |  |
| 203 | | How many births have you ever had births (births that occurred after 28 weeks) (parity)? | In numbers ----------------- |  |
| 204 | | Number of live births | In numbers ----------------- |  |
| 205 | | Number of still births ever had(birth of dead fetus after 28 weeks of gestation) | In numbers ----------------- |  |
| 206 | | Do you have ANC visit in the last pregnancy? | 1 Yes  2 No | If **no** skip to Q. 211 |
| 207 | | If Yes, number of visits | In numbers ----------------- |  |
| 208 | | Where did you attend ANC follow up? | 1. Health Center  2. Hospital  3. Health post |  |
| 209 | | During ANC follow up did you get any information about pregnancy & delivery Complications? | 1. Yes  2. No |  |
| 210 | During ANC follow up, did you get any information about where to deliver? | | 1. Yes  2. No |  |
| 211 | Was your last pregnancy planned? | | 1. Yes  2. No |  |
| 212 | Who is decision maker at home to visit health facility? | | 1.my self  2.husband  3. both  4. other(specify)_________ |  |
| 213 | Have you ever given birth in health institution before the Last/Resent delivery? | | 1. Yes  2. No |  |
| 214 | Where did you deliver your resent birth? | | 1. Health facility  2. Home | **If delivered at facility skip to Q 217** |
| 215 | If birth occurred at **Home**, why did you prefer to deliver at home? | | 1. I feel more comfortable giving birth in home  2. It is my usual practice  3. I don’t like the service in health facilities  4. I have bad experience in giving birth in health facilities  5. Unwelcoming approach of health workers in health facilities  6. The health facility is too far from my house  7. labor was urgent to reach health facilities  8. Lack of money for transport  9. husband influence not to go health facilities  10. family members prefer to give birth in home  11. others reasons, specify-------- |  |
| 216 | Who assisted you during your recent delivery at home? | | 1. No one  2.Health Professionals  3. Trained TBAs  4. Untrained TBAs  5. Health extension worker  6. Family or relatives  7.Others, specify------------------- |  |

| 217 | If birth occurred at health facility, why did you choose that place? | 1. To get better services in health facilities  2. To get better outcomes  3.Bad experience from past home delivery  4.I was informed to deliver in health facilities  5. The health facility closer to my home  6. Others, specify----------------- |  |
| --- | --- | --- | --- |
| 218 | If you gave birth in health facilities, which health facility? | 1.Health center  2.Hospital  3.Private clinic |  |
| 219 | Did your last birth alive or still birth | 1. Alive 2. Still birth |  |

**Part III: knowledge questions related obstetrical danger signs**

| 301 | Have you heard about danger signs of pregnancy and delivery | 1. yes  2. No |  |
| --- | --- | --- | --- |
| 302 | What are the obstetrical danger signs? (Multiple answers possible but do not read the options) | 1. Absent fetal movement  2. Convulsion High blood pressure  3. Blurred vision  4. Severe headache  5. Edema of face/extremities  6. Excessive vaginal bleeding  7. Labour lasting more than 12 hours  8. Retained placenta |  |
